# Design and Performance of a New Zn_0.5_Mg_0.5_FeMnO_4_ Porous Spinel as Anode Material for Li-Ion Batteries

**DOI:** 10.3390/molecules28207010

**Published:** 2023-10-10

**Authors:** Zakaria Chchiyai, Oumayema El Ghali, Abdelilah Lahmar, Jones Alami, Bouchaib Manoun

**Affiliations:** 1 Rayonnement-Matière et Instrumentation, S3M, FST, Hassan First University of Settat, Settat 26000, Morocco; chchiyai.zakaria@gmail.com (Z.C.); oumaimaelghali97@gmail.com (O.E.G.); bouchaib.manoun@um6p.ma (B.M.); 2 Laboratoire de Physique de la Matière Condensée (LPMC), Université de Picardie Jules Verne, 33 rue Saint-Leu, 80039 Amiens, France; 3 Materials Science, Energy, and Nano-Engineering Department, University Mohammed VI Polytechnic, Ben Guerir 43150, Morocco; jones.alami@um6p.ma

**Keywords:** porous Zn_0.5_Mg_0.5_FeMnO_4_ spinel, sol–gel route, crystallographic structure, Li-ion batteries, anode material, electrochemical performance

## Abstract

Due to the low capacity, low working potential, and lithium coating at fast charging rates of graphite material as an anode for Li-ion batteries (LIBs), it is necessary to develop novel anode materials for LIBs with higher capacity, excellent electrochemical stability, and good safety. Among different transition-metal oxides, AB_2_O_4_ spinel oxides are promising anode materials for LIBs due to their high theoretical capacities, environmental friendliness, high abundance, and low cost. In this work, a novel, porous Zn_0.5_Mg_0.5_FeMnO_4_ spinel oxide was successfully prepared via the sol–gel method and then studied as an anode material for Li-ion batteries (LIBs). Its crystal structure, morphology, and electrochemical properties were, respectively, analyzed through X-ray diffraction, high-resolution scanning electron microscopy, and cyclic voltammetry/galvanostatic discharge/charge measurements. From the X-ray diffraction, Zn_0.5_Mg_0.5_FeMnO_4_ spinel oxide was found to crystallize in the cubic structure with *Fd*$\bar{3}$*m* symmetry. However, the Zn_0.5_Mg_0.5_FeMnO_4_ spinel oxide exhibited a porous morphology formed by interconnected 3D nanoparticles. The porous Zn_0.5_Mg_0.5_FeMnO_4_ anode showed good cycling stability in its capacity during the initial 40 cycles with a retention capacity of 484.1 mAh g^−1^ after 40 cycles at a current density of 150 mA g^−1^, followed by a gradual decrease in the range of 40–80 cycles, which led to reaching a specific capacity close to 300.0 mAh g^−1^ after 80 cycles. The electrochemical reactions of the lithiation/delithiation processes and the lithium-ion storage mechanism are discussed and extracted from the cyclic voltammetry curves.

## 1. Introduction

Recently, the extensive demand for consumer electronics and electric vehicles has led to a significant increase in the demand for rechargeable lithium-ion batteries (LIBs) owing to their high energy density, long cycle life, environmental friendliness, and no memory effect [1,2,3]. Since the 1990s, LIBs have been regarded as one of the systems with the most energy storage and have gained widespread applications in mobile electronic devices such as handheld computers, tablets, and mobile phones. LIBs with higher energy density and fast-chargeable capability are requested for many energy storage systems, especially for electric vehicles (EV) and stationary energy storage devices [4,5,6].

In commercialized, traditional LIBs, graphite has been the most extensively used anode material to date due to its low cost and long cycle life [7,8]. Due to the low capacity of graphite material (only 372 mAh g^−1^), its low working potential (<0.2 V vs. Li^+^/Li), and its lithium coating at fast charging rates, graphite-based LIBs are limited and cannot meet the demand for fast-chargeable batteries with high energy density [9,10]. Therefore, developing novel anode materials for LIBs with higher capacity, low cost, nontoxicity, excellent electrochemical stability, and good safety has recently become an important research issue. In this regard, alloys and transition-metal oxides have appeared as the most popular possibilities to substitute the graphite material in LIBs [11,12,13].

In recent decades, transition-metal oxides (TMOs) with the general formula M_x_O_y_ (where M = Mn, Fe, Ni, Sn, Co, Cr, Zn, Nb, Mo, etc.) have received significant attention as promising high-performance anode materials to substitute the graphite material in LIBs due to their distinctive properties such as higher specific capacity than that of the commercially used graphite, good safety, environmental friendliness, and low price [14,15]. With respect to these characteristics, many TMOs have been widely studied as anode materials for LIBs, such as MnO [16], NiO [17], CuO [18], TiO_2_ [19], MnO_2_ [20], SiO_2_ [21], Fe_2_O_3_ [22], and SnO_2_ [23]. Among these TMOs, AB_2_O_4_-type spinels as mixed transition-metal oxides with a greater capacity than graphite anodes (800–1000 mAh g^−1^) have received a lot of attention; they are considered interesting and promising electrochemically active materials for next-generation LIBs, which can combine two transition metals [24,25]. Generally, AB_2_O_4_ spinel oxides as anode materials for LIBs use the conversion mechanism for Li^+^ ion storage [26]. In general, AB_2_O_4_-type spinel oxides with the desired morphology can be easily synthesized through several methods such as solid-state reaction [27], sol–gel [28], co-precipitation [29], hydrothermal [30], electrospinning [31], low-temperature solution combustion [32], microwave-induced combustion [33], and Pechini [34] methods, among others. In recent years, many types of spinel ferrite have been used and emerged as conversion anode materials for LIBs, such as ferrite Fe_3_O_4_ [35], copper ferrite CuFe_2_O_4_ [36], cobalt ferrite CoFe_2_O_4_ [37], nickel ferrite NiFe_2_O_4_ [38], etc. 

Recently, nanostructured spinel ferrites have received tremendous interest for application in LIBs [39]. Among nanostructured spinel ferrites, magnesium ferrite MgFe_2_O_4_ nanoparticles prepared through the sol–gel method, as anode materials for LIBs, have shown a specific capacity near 474 mAh g^−1^ after 50 cycles under a current density of 90 mA g^−1^ [40]. In this phase, Mg was used as an electrochemical inactive pillar to enhance the cyclability of MgFe_2_O_4_ spinel [30]. The manganese ferrite MnFe_2_O_4_ with mesoporous microsphere morphology has been reported by Z. Zhang et al. [41]. It has shown specific capacities of 712.2 and 552.2 mA h g^−1^ at 0.2 C and 0.8 C, respectively, after 50 cycles, suggesting good performance for fast charging. Another spinel ferrite, zinc ferrite ZnFe_2_O_4_, has gained much attention as an anode material for LIBs due to its great electrochemical performance with different morphologies, such as hollow spherical [42], microsized capsules [43], macroporous [44], porous nanospheres [45], and fiber-in-tube [46] morphologies, among others. 

Additionally, manganese-based AB_2_O_4_ spinel oxides have also attracted much interest as alternative electrode materials for LIBs owing to their excellent electrochemical performance [47,48]. Among these manganites, ZnMn_2_O_4_ microsheets were synthesized by K. Cai et al. using a microwave-assisted hydrothermal route [49]. From their electrochemical properties, they showed a capacity of 337.3 mAh g^−1^ after 40 cycles at a rate of 0.1 C. Another spinel manganite, the MgMn_2_O_4_ spinel, was investigated by Z. Wang et al. [50]. The MgMn_2_O_4_ nanoparticles with particle sizes ranging from 20 to 30 nm prepared using the sol–gel method have shown a discharge capacity of 483.6 mAh g^−1^ after 100 cycles.

In recent years, Co-, Ni-, Zn-, Fe-, Cr-, Mn-, and Li-based multi-component spinel oxides have attracted great attention as high-performance anodes for LIBs and long-life lithium-ion batteries [51,52]. L. Dong et al. [51] reported a multi-component transition-metal oxide (Ni,Co,Mn)Fe_2_O_4−x_ with oxygen vacancies as an anode material for LIBs, which has shown a specific capacity of 650.5 mAh g^−1^ at 2 A g^−1^ after 1200 cycles, i.e., excellent reversible capacity and good cycling stability. K. Tian et al. [52] found that spinel oxides (CoNiZnXMnLi)_3_O_4_ (X = Fe, Cr) are high-performance anodes for LIBs. On the other hand, Nb-based oxides such as Nb_2_O_5_, Nb_12_O_29_, TiNb_2_O_7_, Ti_2_Nb_10_O_29_, etc., have attracted much attention as anode materials for high-rate lithium-ion energy storage [53]. 

From the above, to simultaneously improve the stability, energy density, and high-rate capability of anode materials for LIBs, a novel, 3D interconnected structure belonging to transition-metal-oxide-type spinels is urgently needed. In this work, we report a facile route to synthesize 3D porous Zn_0.5_Mg_0.5_FeMnO_4_ spinel oxide using sol–gel method with a theoretical capacity of 976.3 mAh g^−1^ as an anode electrode for LIBs. Herein, Mg is used as an inactive stabilized pillar, which can produce good cycling stability. The synthesized active material is characterized by different techniques such as X-ray diffraction, thermogravimetry analysis, scanning electron microscopy, and the nitrogen adsorption–desorption isotherm to report, respectively, its crystallographic structure, thermal stability, morphology, and specific surface area. The electrochemical performance of the prepared porous Zn_0.5_Mg_0.5_FeMnO_4_ spinel oxide is tested by cyclic voltammetry and galvanostatic discharge/charge measurements.

## 2. Results and Discussion

### 2.1. X-ray Diffraction, Rietveld Refinement, and Crystal Structure

**Figure 1:**
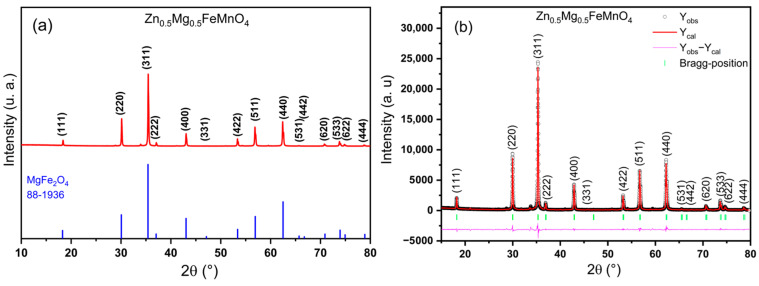
(**a**) X-ray powder diffraction pattern of the elaborated Zn_0.5_Mg_0.5_FeMnO_4_ spinel oxide compared with JCPDS card no. 88–1936 of the MgFe_2_O_4_ phase. (**b**) Rietveld refinement of the XRD pattern for the elaborated Zn_0.5_Mg_0.5_FeMnO_4_ spinel oxide with the cubic *Fd*$\bar{3}$*m* structure.

The X-ray powder diffraction (XRD) technique was first employed to analyze the crystallographic structure, formation, and phase purity of the as-synthesized Zn_0.5_Mg_0.5_FeMnO_4_ sample. Figure 1a shows the wide-angle XRD pattern of the Zn_0.5_Mg_0.5_FeMnO_4_ sample acquired at room temperature. From this figure, the XRD data reveal the formation of the AB_2_O_4_-type spinel phase as all the experimental XRD peaks observed at different 2θ values are well matching with standard peaks of the MgFe_2_O_4_ spinel phase that crystallizes in the cubic *Fd*$\bar{3}$*m* structure with a lattice parameter of a = 8.3827 Å (JCPDS card: 88-1936) [54], which indicates that the as-synthesized Zn_0.5_Mg_0.5_FeMnO_4_ sample exhibits a single spinel phase with the cubic *Fd*$\bar{3}$*m* system. However, no additional peaks from any impurities could be identified in the XRD pattern, revealing a high purity of the synthesized spinel phase and confirming that the organic species of CA have been completely removed. In addition, the synthesized spinel shows sharp XRD peaks with small full width at half maximum (FWHM), suggesting good crystallization of the synthesized Zn_0.5_Mg_0.5_FeMnO_4_ spinel oxide. Besides, the widening of the obtained XRD peaks provides proof of the existence of nanoscale-dimension spinel particles.

For further deep analysis of structural parameters, the XRD pattern of the synthesized Zn_0.5_Mg_0.5_FeMnO_4_ spinel ferrite was refined using the Rietveld method [55,56]. The structural refinement was carried out with the cubic structure and *Fd*$\bar{3}$*m* as a space group. The Rietveld refinement plot of the synthesized Zn_0.5_Mg_0.5_FeMnO_4_ spinel is displayed in Figure 1b, where the Bragg reflections are revealed by the green vertical bars, the observed XRD (Y_obs_) pattern is shown by open black circles, the simulated XRD (Y_cal_) pattern by the Rietveld method is represented by red solid lines, and the difference curve (Y_obs_–Y_cal_) is indicated by pink solid lines. From this figure, it can be seen that the observed XRD pattern shows great agreement with the calculated XRD pattern, revealing the success of a realized structural refinement for the synthesized Zn_0.5_Mg_0.5_FeMnO_4_ spinel ferrite. Moreover, the quality of the structural refinement was evaluated by the consideration of the different R-factors such as structure factor (R_F_), Bragg factor (R_B_), profile factor (R_P_), expected factor (R_exp_), and profile factor (R_wp_) [57,58,59,60]. The results of the powder X-ray diffraction of the synthesized Zn_0.5_Mg_0.5_FeMnO_4_ spinel oxide in the Rietveld refinement such as structural parameters, profile parameters, and different reliability R-factors are summarized in Table 1. According to the obtained values of different R-factors, the synthesized Zn_0.5_Mg_0.5_FeMnO_4_ spinel oxide crystallizes in the cubic *Fd*$\bar{3}$*m* structure with a lattice parameter of a = 8.4265 (2) Å.

**Figure 2:**
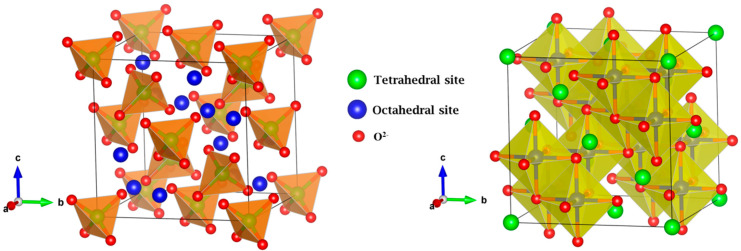
Three-dimensional crystal structure of Zn_0.5_Mg_0.5_FeMnO_4_ spinel oxide with the cubic *Fd*$\bar{3}$*m* structure. The Zn^2+^ and Mg^2+^ cations are located in the tetrahedral sites (green balls), Fe^3+^ and Mn^3+^ cations are located in the octahedral sites (blue balls), and the red balls represent the O^2−^ oxygen anions.

From the obtained structural parameters by Rietveld refinement, the crystal structure of Zn_0.5_Mg_0.5_FeMnO_4_ spinel oxide was drawn. Figure 2 displays the crystal structure of Zn_0.5_Mg_0.5_FeMnO_4_ spinel oxide with the cubic structure in *Fd*$\bar{3}$*m* symmetry. As clearly shown in the figure, the crystal structure of Zn_0.5_Mg_0.5_FeMnO_4_ spinel oxide is constructed by 3D interconnected tetrahedral and octahedral polyhedra, where Zn^2+^ and Mg^2+^ cations are located in the tetrahedral sites (green balls), Fe^3+^ and Mn^3+^ are located in the octahedral sites (blue balls), and the red balls represent the O^2−^ oxygen anions. Moreover, this structure shows three Wyckoff sites—the 8a, 16d, and 32e sites, which correspond to the tetrahedral, octahedral, and oxygen sites, respectively. Only 1/8 of the tetrahedral sites are occupied by Zn^2+^/Mg^2+^ cations, 1/2 of the octahedral sites are occupied by Fe^3+^/Mn^3+^ cations, and the 32e sites are totally occupied by oxygen anions. Therefore, the spinel structure exhibits 7/8 of the tetrahedral sites and 1/2 of the octahedral sites that are empty. During the first discharge of electrochemical cells, the lithium ions can be inserted into the unit cell to occupy the lacunar sites.

### 2.2. Thermal Analysis

**Figure 3:**
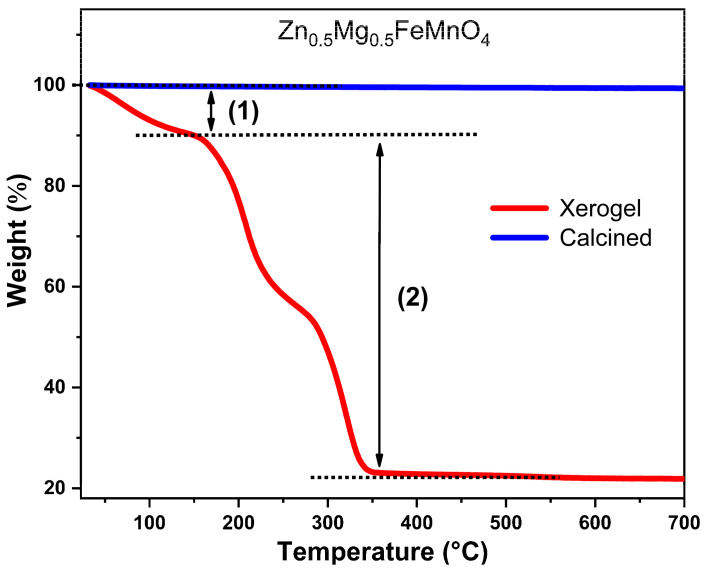
Thermogravimetric curves (TG) of the synthesized xerogel and calcined sample of Zn_0.5_Mg_0.5_FeMnO_4_ spinel.

In order to understand the mechanism of the calcination of the synthesized xerogel, the xerogel sample was characterized by thermogravimetric analysis (TGA) in a wide temperature range of 20–700 °C. Figure 3 depicts the thermogravimetric curves (TG) of the synthesized xerogel and the calcined sample of Zn_0.5_Mg_0.5_FeMnO_4_ spinel. In the case of the xerogel sample (red curve), two weight losses take place in the temperature range from room temperature to 350 °C. The first loss is observed before 140 °C, which is due to the evaporation of the water used as a solvent during the synthesis and the physically adsorbed water on the surface. The second weight loss observed in the temperature range of 140–350 °C is attributed to the decomposition of the organic species of the citric acid (CA) associated with the elimination of nitrogen dioxide (NO_2_^↑^). Further, no weight loss is present above 350 °C. Moreover, the thermal stability of the synthesized spinel was examined by thermogravimetric analysis (TGA). For the calcined sample at 700 °C (blue curve), its TG curve demonstrates no weight loss in the whole temperature range of 20–700 °C, indicating good thermal stability of the synthesized porous Zn_0.5_Mg_0.5_FeMnO_4_ spinel oxide and confirming that the organic molecules of the citric acid (CA) have been completely removed after calcination at 700 °C.

### 2.3. Morphology, Purity, and Specific Surface

**Figure 4:**
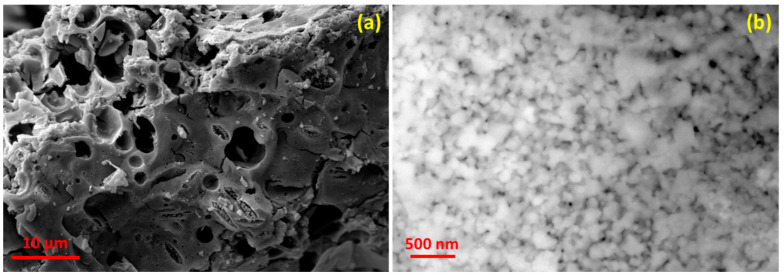
(**a**) Scanning electron microscopy (SEM) image of the synthesized porous Zn_0.5_Mg_0.5_FeMnO_4_ spinel oxide and (**b**) its high-resolution scanning electron microscopy (HR-SEM) image.

The microstructural morphology of the synthesized spinel was revealed by scanning electron microscopy (SEM). Figure 4a shows the SEM image of the synthesized Zn_0.5_Mg_0.5_FeMnO_4_ spinel oxide, which demonstrates its surface morphology. From the figure, it can be seen that the prepared spinel shows a microsized porous morphology with different pore sizes ranging from 2 to 5 μm. Moreover, the porous morphology can make the electrolyte used in the cells of LIBs penetrate easily, which leads to the acceleration of lithium ions diffusion, enhancing the electrochemical performance. For further deep analysis of the surface morphology, the synthesized spinel was characterized by high-resolution scanning electron microscopy (HR-SEM). The obtained HR-SEM image is presented in Figure 4b. It can be seen from this figure that the porous Zn_0.5_Mg_0.5_FeMnO_4_ spinel is composed of interconnected nanosized spherical particles with sizes ranging from 50 to 100 nm.

**Figure 5:**
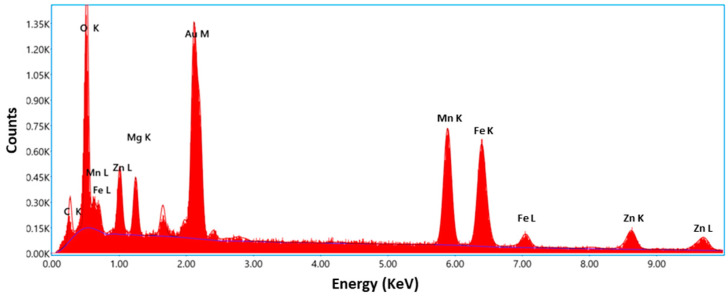
EDX spectrum of the synthesized porous Zn_0.5_Mg_0.5_FeMnO_4_ spinel oxide.

**Figure 6:**
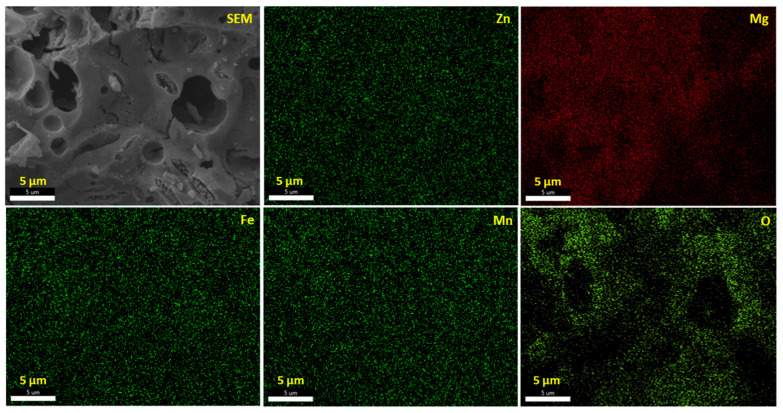
The SEM image and EDX element mapping results of Zn, Mg, Fe, Mn, and O of the synthesized porous Zn_0.5_Mg_0.5_FeMnO_4_ spinel oxide. Scale bar, 5 μm.

To carry out an investigation on the purity of the synthesized porous Zn_0.5_Mg_0.5_FeMnO_4_ spinel oxide, an energy-dispersive X-ray (EDX) spectrometer was utilized. The results of this investigation are displayed in Figure 5. As clearly shown in the figure, only Zn, Mg, Fe, Mn, and O peaks could be recorded, which suggests the existence of only Zn, Mg, Fe, Mn, and O elements in the product. No additional element was recorded. The EDX results confirm approximately the atomic ratio 0.5:0.5:1:1:4 of Zn, Mg, Fe, Mn, and O elements, respectively, which gives further proof of the high purity of the synthesized spinel. Besides, the SEM/EDX instrument was also used to provide different element distribution maps of the 3D metal oxide network. The surface of the synthesized spinel and its EDX element mapping results are shown in Figure 6. From these maps, it is clearly demonstrated that the Zn, Mg, Fe, Mn, and O elements are uniformly distributed across the whole sample.

**Figure 7:**
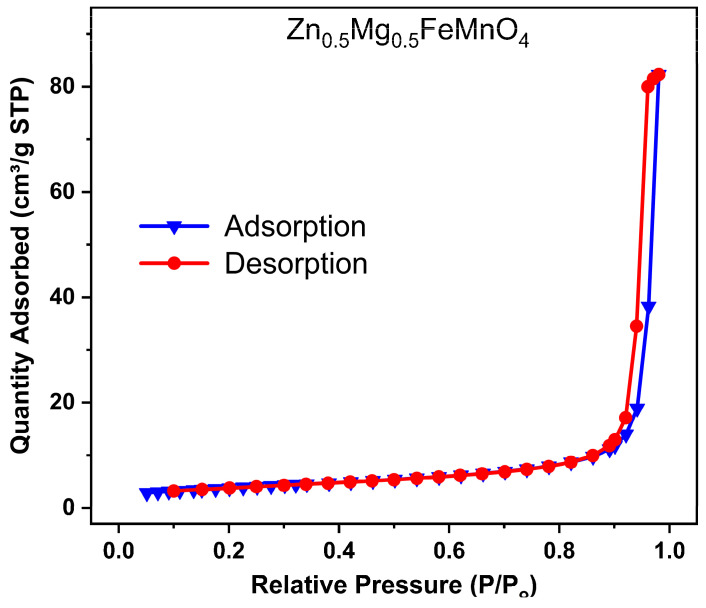
Nitrogen adsorption–desorption isotherm of the synthesized porous Zn_0.5_Mg_0.5_FeMnO_4_ spinel oxide.

Figure 7 shows the nitrogen adsorption–desorption isotherm of the synthesized porous Zn_0.5_Mg_0.5_FeMnO_4_ spinel oxide collected at a liquid nitrogen temperature of 77 K. The analysis of the nitrogen adsorption–desorption isotherm was performed to obtain the specific surface area of the synthesized porous Zn_0.5_Mg_0.5_FeMnO_4_ spinel oxide using the Brunauer–Emmett–Teller (BET) method. From the nitrogen adsorption–desorption isotherm, the porous spinel shows a type IV nitrogen sorption isotherm with a small hysteresis loop at high partial pressures (P/P_0_), suggesting a characteristic of a mesoporous material. Using the Brunauer–Emmett–Teller (BET) method, the specific surface area of the synthesized porous Zn_0.5_Mg_0.5_FeMnO_4_ spinel oxide was found to be 13.54 m^2^ g^−1^. The obtained small specific surface area is probably due to the high aggregation of nanoparticles, as shown in the HR-SEM image (Figure 4b). Moreover, the synthesized porous Zn_0.5_Mg_0.5_FeMnO_4_ spinel oxide has a larger specific surface area than that of graphite powder, which is 6.89 m^2^ g^−1^ [61]. 

### 2.4. Electrochemical Results

**Figure 8:**
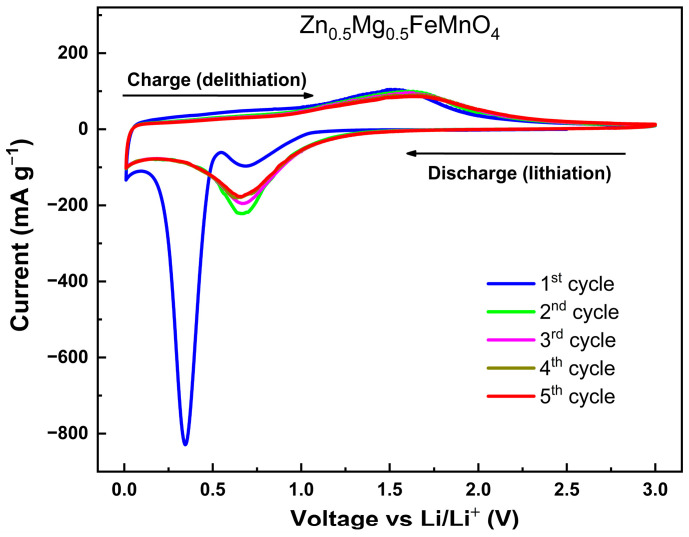
Cyclic voltammetry (CV) curves of the synthesized porous Zn_0.5_Mg_0.5_FeMnO_4_ spinel oxide measured over the 0.01–3.00 V voltage range (vs. Li^+^/Li) at a scan rate of 0.05 mV s^−1^ for the initial five cycles.

In order to further investigate the beneficial effects of the Zn_0.5_Mg_0.5_FeMnO_4_ spinel oxide with a porous morphology as anode for LIBs, its electrochemical performance was analyzed in a half-cell configuration. The cyclic voltammetry (CV) measurements were conducted over the 0.01–3.00 V voltage range vs. Li^+^/Li under ambient conditions. Figure 8 shows the initial five CV curves of the synthesized porous Zn_0.5_Mg_0.5_FeMnO_4_ spinel oxide collected at a sweep rate of 0.05 mV s^−1^. Specifically, in the initial cathodic sweep (discharge, lithiation), two dominant peaks at 0.35 and 0.7 V are observed in the first cycle. In agreement with previous studies [62,63], the cathodic peak that appeared at 0.7 V is attributed to the reversible reduction of Zn^2+^, Fe^3+^, and Mn^3+^ ions together, which are incorporated in Zn_0.5_Mg_0.5_FeMnO_4_ spinel phase to their metallic states Zn, Fe, and Mn associated with the formation of MgO and amorphous Li_2_O, as demonstrated in Equation (1). After the reduction of Zn_0.5_Mg_0.5_FeMnO_4_, the formed Zn metal can react with Li^+^ ions, giving the reversible formation of LiZn alloys (Equation (2)) [46]. The other intense peak observed at 0.35 V corresponds to electrolyte decomposition, which leads to the irreversible formation of a solid electrolyte interface (SEI) [64,65]. For the subsequent cathodic sweeps, only one peak is obtained near 0.67 V, which is assigned to the reduction of ZnO, Fe_2_O_3_, and MnO to Zn, Fe, and Mn, respectively, as previously reported [66,67]. The electrochemical reactions of the lithiation process (discharge) can be shown, as reported in [68,69].

- First cycle:

Zn_0.5_Mg_0.5_FeMnO_4_ + 7 Li^+^ + 7 e^−^ → 0.5 Zn + 0.5 MgO + Fe + Mn + 3.5 Li_2_O (1)

Zn + Li^+^ + e^−^   →   LiZn (2)

- Subsequent cycles:

0.5 ZnO + 0.5 Fe_2_O_3_ + MnO + 6 Li^+^ + 6 e^−^   →   0.5 Zn + Fe + Mn + 3 Li_2_O (3)

In the charge process (delithiation), the distinct anodic peaks are between 1.6 V and 1.8 V for all cycles, which probably contribute to the various oxidations of metallic Zn, Fe, and Mn to their oxide states Zn^2+^ (ZnO), Fe^3+^ (Fe_2_O_3_), and Mn^2+^ (MnO), respectively, as represented in Equation (4) [39,70]. Therefore, the related reactions of the charge process (delithiation) for all cycles can be summarized as follows [71,72]:

0.5 Zn + Fe + Mn + 3 Li_2_O     →     0.5 Zno + 0.5 Fe_2_O_3_ + MnO + 6 Li^+^ + 6 e^−^ (4)

LiZn    →    Zn + Li^+^ + e^−^ (5)

On the other hand, the first cycle is easily distinct from the subsequent cycles. Besides, the difference between the redox peaks in the first and subsequent cycles is due to the capacity loss of the anode spinel during the first cycle. From the third cycle, the CV curves are similar and the redox peaks remain consistent, revealing good reversibility of the Zn_0.5_Mg_0.5_FeMnO_4_ anode.

**Figure 9:**
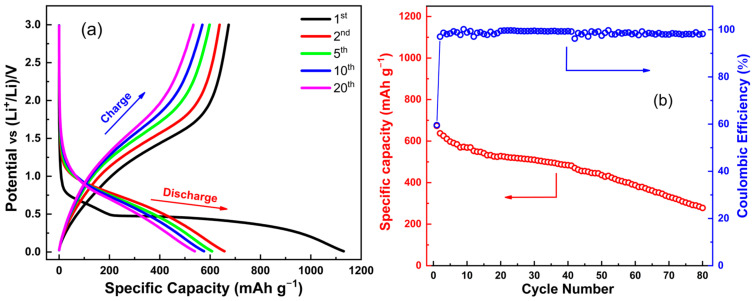
Electrochemical analyses of the synthesized porous Zn_0.5_Mg_0.5_FeMnO_4_ spinel oxide: (**a**) selected galvanostatic discharge/charge curves measured over the 0.01–3.00 V voltage range (vs. Li^+^/Li) at an applied current density of 0.15 A g^−1^ for various cycles (1st, 2nd, 5th, 10th, and 20th) and (**b**) cycling stability in its specific capacity and Coulombic efficiency during 80 cycles.

Subsequently, the results of the galvanostatic discharge/charge cycling of the synthesized porous Zn_0.5_Mg_0.5_FeMnO_4_ spinel oxide acquired at a current density of 150 mA g^−1^ over the potential region of 0.01–3.00 V (vs. Li^+^/Li) for various cycles (1st, 2nd, 5th, 10th, and 20th) are shown in Figure 9a. In accordance with previous reports, the profile of the potential versus capacity plot of the studied porous Zn_0.5_Mg_0.5_FeMnO_4_ spinel oxide is similar to those of ZnFe_2_O_4_ and MgFe_2_O_4_ [73]. It is worth recalling that the Zn_0.5_Mg_0.5_FeMnO_4_ spinel oxide has a theoretical capacity of 976.3 mAh g^−1^ as an anode electrode for LIBs. From Figure 9a, the initial discharge and charge capacities are 1132.9 and 676.4 mA h g^−1^, respectively. Note that the initial discharge capacity is higher than the theoretical capacity. This can be explained by the lithium-ion storage through a surface faradic redox reaction (pseudocapacitive). The irreversible capacity loss between discharge and charge of the first cycle is normally due to the formation of SEI film [74,75]. The studied spinel displays an extended voltage plateau at about 0.5 V followed by a gradual slope for the first discharge. The potential plateau is probably due to the conversion from the reduction of Zn^2+^, Fe^3+^, and Mn^3+^ transition metals to metallic states of Zn, Fe, and Mn, respectively [76,77]. During discharge/charge cycling, the discharge and charge capacities gradually decrease with increasing cycle numbers, which is probably attributed to the aggregation and pulverization of Zn_0.5_Mg_0.5_FeMnO_4_ nanoparticles, leading to poor electrical contacts between Zn_0.5_Mg_0.5_FeMnO_4_ nanoparticles and that between the active material and current collector [40]. The cycling performance of the Zn_0.5_Mg_0.5_FeMnO_4_ anode at the current density of 150 mA g^−1^ during 80 cycles is displayed in Figure 9b. It is clearly seen that the Zn_0.5_Mg_0.5_FeMnO_4_ anode shows good cycling stability in its capacity during the initial 40 cycles, with a specific capacity of 484.1 mAh g^−1^ after 40 cycles followed by a gradual decrease in the range of 40–80 cycles. Moreover, the Zn_0.5_Mg_0.5_FeMnO_4_ anode exhibits a specific capacity close to 300.0 mAh g^−1^ after 80 cycles at an applied current density of 150 mA g^−1^. For the first cycle, the anode gives a Coulombic efficiency of 60.2%, which is owing to the abovementioned capacity loss in the first cycle. From the second cycle, the anode provides a stable value of Coulombic efficiency, which is more than 98% during 80 cycles. In order to compare the obtained performance with the literature, Table 2 compares the results of the electrochemical performance of the elaborated Zn_0.5_Mg_0.5_FeMnO_4_ spinel oxide with previously studied anodes.

**Figure 10:**
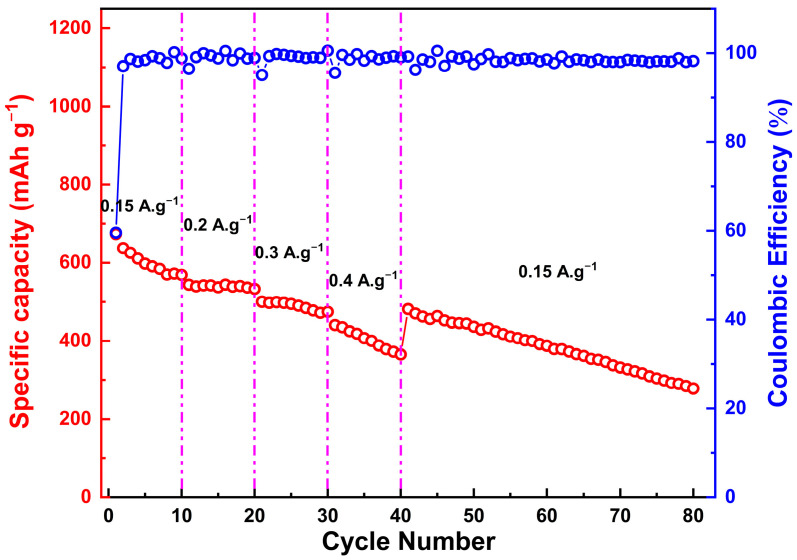
Rate performance analysis of the synthesized porous Zn_0.5_Mg_0.5_FeMnO_4_ spinel oxide measured over the 0.01–3.00 V voltage range (vs. Li^+^/Li) with step-wise increasing current rates of 0.15, 0.2, 0.3, and 0.4 A g^−1^.

As presented in Figure 10, the rate performance of the studied Zn_0.5_Mg_0.5_FeMnO_4_ anode is examined by increasing the scan rates from 150 to 400 mA g^−1^ and then back to 150 mA g^−1^. For the first current density (150 mA g^−1^), the Zn_0.5_Mg_0.5_FeMnO_4_ anode reaches a specific capacity of 569.1 mAh g^−1^ after 10 cycles. When gradually increasing the current density, the specific capacity of the Zn_0.5_Mg_0.5_FeMnO_4_ anode gradually decreases and reaches a specific capacity of 533.2 mAh g^−1^ after 20 cycles at a current density of 200 mA g^−1^, and a specific capacity of 474.6 mAh g^−1^ after 30 cycles at a current density of 300 mA g^−1^, with good cycling stability. For the subsequent 10 cycles, the Zn_0.5_Mg_0.5_FeMnO_4_ anode exhibits a specific capacity of 365.3 mAh g^−1^ after 40 cycles at a current density of 400 mA g^−1^. When the current density is switched back to 150 mA g^−1^, the specific capacity of the studied anode increased rapidly to reach 481.8 mAh g^−1^. In the range of 40–80 cycles, the Zn_0.5_Mg_0.5_FeMnO_4_ anode was found to show a gradual decrease during discharge/charge cycling and reached a specific capacity close to 300.0 mAh g^−1^ after 80 cycles at an applied current density of 150 mA g^−1^.

**Figure 11:**
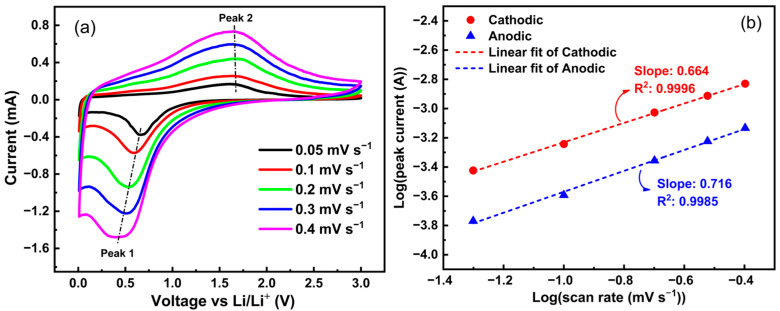
(**a**) Cyclic voltammetry (CV) curves of the synthesized porous Zn_0.5_Mg_0.5_FeMnO_4_ spinel oxide at scan rates of 0.05, 0.1, 0.2, 0.3, and 0.4 mV s^−1^. (**b**) The determination of the *b*-value of the synthesized porous Zn_0.5_Mg_0.5_FeMnO_4_ spinel oxide using the linear fitting of the peak currents versus scan rates.

For further deep analysis of the kinetics of electrochemical lithiation/delithiation processes, the CV curves of the studied Zn_0.5_Mg_0.5_FeMnO_4_ anode were acquired at various scan rates ranging from 0.05 to 0.40 mV s^−1^, as shown in Figure 11a. The measured peak current (*i*) for the cathodic and anodic sweeps at various scan rates (*v*) was used to analyze the lithium-ion intercalation kinetics of the studied Zn_0.5_Mg_0.5_FeMnO_4_ anode. For the cathodic and anodic sweeps, the peak current (*i*, A) and the scan rates (*v*, mV s^−1^) can be related according to the Dunn relation, as expressed in Equation (6) [79,80]:

*i* = *aν^b^* (6)

Both sides of Equation (6) were taken in the logarithm function, which can be written as

Log(*i*) = Log(*a*) + *b* Log(*ν*) (7)

In these expressions, *a* and *b* are adjustable parameters. In general, three lithium-ion intercalation kinetics can be distinguished depending on the *b*-value. In particular, when the *b*-value approaches 0.5, the lithium-ion storage mechanism is controlled by the diffusion process. When the *b*-value is close to 1, the lithium-ion storage mechanism is through a surface faradic redox reaction (pseudocapacitive); whereas, when the *b*-value is between 0.5 and 1, the lithium-ion storage mechanism is a combination of a pseudocapacitive process and diffusion-controlled insertion [81]. The *b*-value was determined by plotting Log(*i*) against Log(*v*) for cathodic and anodic peaks, as shown in Figure 11b. The *b*-value for the cathodic and anodic sweeps corresponds to the slope of linear fitting of the peak currents versus scan rates. According to the estimated slopes (Figure 11b), the lithium-ion storage mechanism of the studied Zn_0.5_Mg_0.5_FeMnO_4_ anode is a combination of diffusion-controlled intercalation and pseudocapacitance behavior.

**Figure 12:**
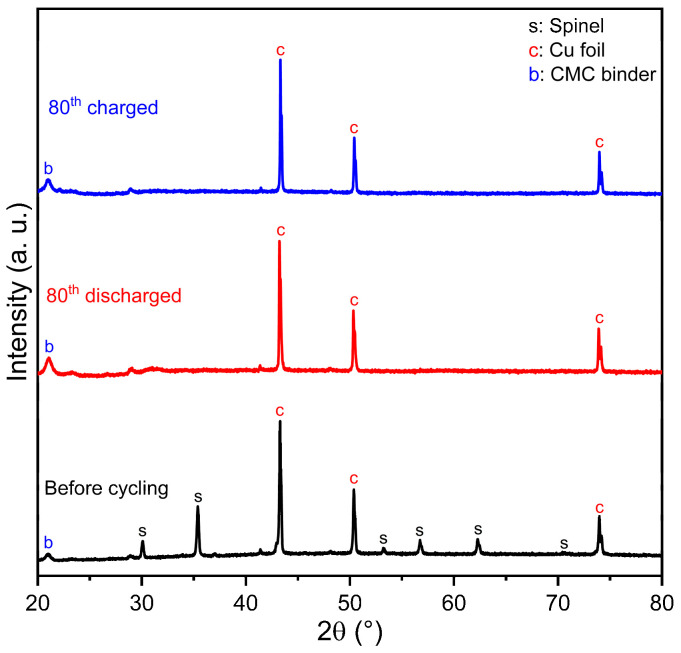
Ex-XRD patterns of the synthesized Zn_0.5_Mg_0.5_FeMnO_4_ electrodes in different states: before cycling, discharged, and charged after 80 cycles.

For further study of the changes in the structure and morphology of the electrode material after cycling, and to reveal the lithium storage mechanism, ex situ XRD and SEM characterizations of the uncycled and cycled electrodes at a current density of 0.15 A g^−1^ were explored. Figure 12 shows the ex-XRD (20–80°) patterns of the synthesized Zn_0.5_Mg_0.5_FeMnO_4_ electrodes in different states: before cycling, discharged, and charged after 80 cycles. From this figure, the XRD pattern of the synthesized Zn_0.5_Mg_0.5_FeMnO_4_ electrode before cycling confirms the presence of the spinel phase, Cu foil, and the used CMC as a binder. After the discharge process, all the diffraction peaks of the Zn_0.5_Mg_0.5_FeMnO_4_ spinel phase disappear, revealing that the Zn_0.5_Mg_0.5_FeMnO_4_ spinel became an amorphous phase. Subsequently, there is no added peak observed after the charge process, revealing that it still maintains an amorphous phase under continuous lithiation/delithiation processes, which was an interesting discovery. Similar results have been obtained in several previous works that studied transition-metal-based spinels as anodes for LIBs [82,83]. According to C. Duan et al. [84], the amorphous phase formed after the first discharge is beneficial to buffer the volume change of the electrode and maintain the stability and integrity of the electrode. 

**Figure 13:**
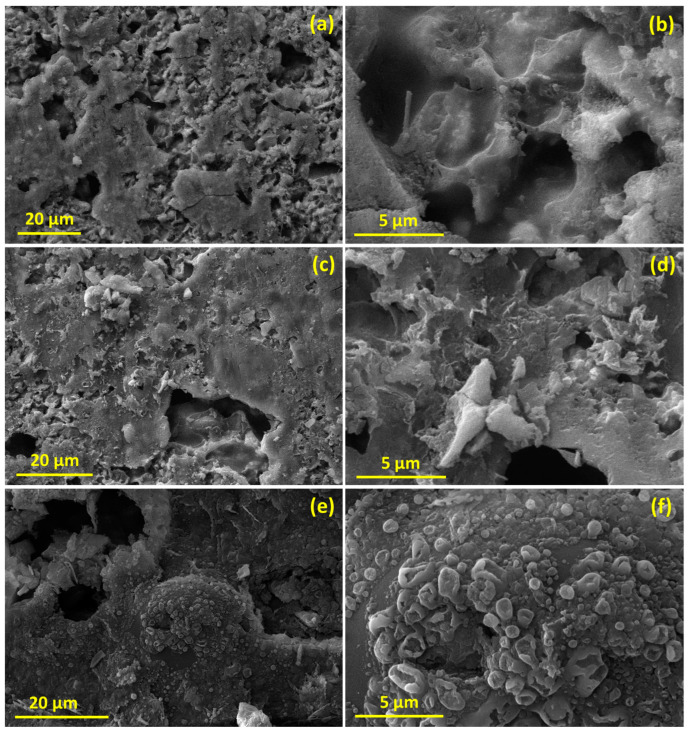
SEM images of the synthesized Zn_0.5_Mg_0.5_FeMnO_4_ electrodes (**a**,**b**) before cycling, (**c**,**d**) discharged after 80 cycles, and (**e**,**f**) charged after 80 cycles.

To demonstrate the microstructural change in the studied electrode after cycling, the SEM images of the synthesized Zn_0.5_Mg_0.5_FeMnO_4_ electrodes in different states—before cycling, discharged, and charged after 80 cycles—are displayed in Figure 13. Compared to the state before cycling, the Zn_0.5_Mg_0.5_FeMnO_4_ electrode shows significant spherical porosity constructed by 3D interconnected nanoparticles. After 80 cycles, it can be seen that the porosity of the synthesized Zn_0.5_Mg_0.5_FeMnO_4_ spinel is diminished and the surface becomes dense, whereas the size of the nanoparticles increased obviously due to the huge volume change during continuous lithiation/delithiation processes. This confirms the aggregation of Zn_0.5_Mg_0.5_FeMnO_4_ nanoparticles, which led to a reduction in the specific surface area. This reduction in the specific surface area can explain the gradual decrease and poor stability of the specific capacity during discharge/charge processes with cycling. 

## 3. Materials and Methods

### 3.1. Materials

Zinc chloride ZnCl_2_, magnesium nitrate hexahydrate Mg(NO_3_)_2_·6H_2_O, iron nitrate nonahydrate Fe(NO_3_)_3_·9H_2_O, manganese nitrate tetrahydrate Mn(NO_3_)_2_·4H_2_O, and citric acid monohydrate C_6_H_8_O_7_·H_2_O of analytical grade were purchased from Sigma Aldrich. All purchased reagents were directly used as received without further purification. 

### 3.2. Synthesis of the Porous Zn_0.5_Mg_0.5_FeMnO_4_ Spinel

The porous Zn_0.5_Mg_0.5_FeMnO_4_ spinel oxide was synthesized using a facile sol–gel method. Initially, specific molar ratios of ZnCl_2_, Mg(NO_3_)_2_·6H_2_O, Fe(NO_3_)_3_·9H_2_O, and Mn(NO_3_)_2_·4H_2_O were dissolved together in deionized water under vigorous magnetic stirring for 30 min to obtain a homogeneous solution. To this homogeneous solution, citric acid (CA) dissociated in deionized water was added at a molar ratio of CA/cations = 2.5. Then, the solution was kept on the hot plate at 90 °C until a viscous gel formed followed by cooling slowly down to room temperature. Subsequently, the resulting gel was dried in an oven at 120 °C for 12 h to obtain the xerogel powder. Finally, the as-synthesized powder was annealed in the furnace at 700 °C for 12 h at a heating rate of 3 °C min^−1^ in air to obtain the porous Zn_0.5_Mg_0.5_FeMnO_4_ spinel oxide. During this synthesis, the Zn_0.5_Mg_0.5_FeMnO_4_ spinel oxide was formed from the reagents according to the following chemical reaction:

0.5 ZnCl_2_ + 0.5 Mg(NO_3_)_2_·6H_2_O + Fe(NO_3_)_3_·9H_2_O + Mn(NO_3_)_2_·4H_2_O → Zn_0.5_Mg_0.5_FeMnO_4_ + 0.5 Cl_2_^↑^ + 6 NO_2_^↑^ + 16 H_2_O^↑^

### 3.3. Characterization Techniques

For the structural analysis and the phase identification, the synthesized porous Zn_0.5_Mg_0.5_FeMnO_4_ spinel was firstly characterized by powder X-ray diffraction (XRD, Bruker D8 Advance) with Cu-K_α_ (λ_1_ = 1.54056 Å and λ_2_ = 1.54439 Å) radiations. The XRD analysis was carried out at room temperature over a 2 theta range from 10° to 80° at a scan rate of 0.02°/s. The structural refinement of the prepared sample was carried out by the Rietveld method using Full-prof software (version 7.95) [85]. Thermal stability of the synthesized xerogel and calcined sample was investigated by thermogravimetric analysis (TGA) performed on a Discovery TGA analyzer in the temperature range of 20–700 °C at a heating rate of 10 °C min^−1^ under a constant air purging flow. The surface morphology and surface elemental composition of the prepared powder spinel were evaluated by employing a scanning electron microscope interconnected with an energy-dispersive X-ray spectrometer (SEM/EDX, Zeiss Evo 10). For further deep analysis of the surface morphology, a high-resolution scanning electron microscopy (HR-SEM) image was obtained on a JEOL JSM-IT500HR instrument. To obtain the Brunauer–Emmett–Teller (BET) specific surface area of the synthesized porous spinel, the nitrogen adsorption–desorption isotherm was collected at a liquid nitrogen temperature of 77 K using a Micromeritics Instrument.

### 3.4. Electrochemical Measurements

To make the working electrodes of Zn_0.5_Mg_0.5_FeMnO_4_ spinel, dry powders of the synthesized spinel (active material), carbon black, and carboxymethyl cellulose (CMC) binder were mixed at a weight ratio of 80:10:10, respectively. To form a homogeneous slurry, the mixture was dispersed in ultrapure water and mixed for 2 h under vigorous magnetic stirring. Subsequently, the above slurry mixture was cast on a copper foil current collector with a thickness of 0.1 mm and a mass loading of 1~1.2 mg cm^−2^ using the doctor-blade technique. The loaded copper foil was dried at 60 °C under vacuum for 4 h to eliminate the water used as a solvent and then cut into disks of 11 mm in diameter. The produced electrodes were dried at 80 °C under vacuum for 12 h. All the electrochemical measurements of Zn_0.5_Mg_0.5_FeMnO_4_ spinel as anode material for LIBs were performed at room temperature using CR2032 coin cells in a half-cell configuration. Zn_0.5_Mg_0.5_FeMnO_4_//Li coin cells were assembled in an Ar (99.999%)-filled glove box (Jacomex, France, H_2_O and O_2_ contents ≤ 1.0 ppm) with high-purity metallic lithium disks as the counter and reference electrodes, Whatman as a separator, and a solution of LiPF_6_ (2 mol L^−1^) dissolved in EMC as the electrolyte. Their electrochemical performance was examined using a BioLogic Science multi-channel battery cycler for testing cyclic voltammetry (CV), galvanostatic discharge/charge, long-term cycling, and rate capability over the 0.01–3.00 V voltage range (vs. Li^+^/Li). The cyclic voltammetry (CV) data were measured at various scan rates: 0.05, 0.10, 0.20, 0.30, and 0.40 mV s^−1^. The galvanostatic discharge/charge and long-term cycling of the studied spinel were performed at a current density of 150 mA g^−1^. Moreover, the rate capability analysis was realized at different current densities of 150, 200, 300, and 400 mA g^−1^. The assembled coin cells were set still for 7 h before testing. In this work, the discharging and charging are considered as the lithiation and delithiation processes, respectively.

## 4. Conclusions

In this work, the electrochemical performance of a porous Zn_0.5_Mg_0.5_FeMnO_4_ spinel oxide elaborated by a facile sol–gel route as anode material for LIBs was investigated in detail. As demonstrated by the XRD, Rietveld method, SEM, and EDX results, the product has a high-purity phase with the desired chemical composition. From the structural analysis, the formation of a single AB_2_O_4_-type spinel phase of the synthesized sample was confirmed. Rietveld refinement evidenced the cubic spinel with *Fd*$\bar{3}$*m* symmetry. Under flowing air, the synthesized porous Zn_0.5_Mg_0.5_FeMnO_4_ spinel oxide showed a good thermal stability as the temperature increased to 700 °C. The SEM image confirmed the successful formation of porous morphology with pore sizes ranging from 2 to 5 μm. From the HR-SEM image, the 3D interconnection of the nanosized spherical particles led to a porous morphology. According to the BET method, the specific surface area of the synthesized porous Zn_0.5_Mg_0.5_FeMnO_4_ spinel oxide was found to be 13.54 m^2^ g^−1^. For the electrochemical performance, the Zn_0.5_Mg_0.5_FeMnO_4_ anode showed good cycling stability in its capacity during the initial 40 cycles, with a retention capacity of 484.1 mAh g^−1^ after 40 cycles at a current density of 150 mA g^−1^, followed by a gradual decrease in the range of 40–80 cycles, which led to reaching a specific capacity close to 300.0 mAh g^−1^ after 80 cycles at a current density of 150 mA g^−1^. Besides, the electrochemical reactions of the lithiation/delithiation processes were extracted from the CV curves. For the lithium-ion storage mechanism, a combination of diffusion-controlled intercalation and pseudocapacitance behavior was found. In general, this work is a way forward to develop inexpensive, highly efficient, and eco-friendly transition-metal oxide as a promising anode material for LIBs.

## Figures and Tables

**Table 1:** The results of the Rietveld structural refinement for structural parameters, profile parameters, and various reliability R-factors of the elaborated Zn_0.5_Mg_0.5_FeMnO_4_ spinel oxide.

**Wavelengths (Å)**	Lattice Parameters	Crystal Structure	Mixing Factor	Caglioti Parameters	R-Factors
λ_kα1_ = 1.54056 λ_kα2_ = 1.54439	a = 8.4265 (2) Å V = 598.33 (1) Å^3^	Cubic *Fd*$\bar{3}$*m*	η = 635 (7)	U = 0.029 (6) V = −0.011 (6) W = 0.0154 (2)	R_F_ = 2.41, R_B_= 2.83 R_P_ = 7.27, R_exp_ = 11.5 R_wp_ = 12.3

**Table 2:** The results of the electrochemical performance of the elaborated Zn_0.5_Mg_0.5_FeMnO_4_ spinel oxide compared with previously studied anodes.

Anode	Applied Current Density (mA g^−1^)	Cycle Number	Specific Capacity (mAh g^−1^)	Ref.
Graphite	76	50	265	[78]
MgFe_2_O_4_ nanoparticles	90	50	474	[40]
MnFe_2_O_4_ mesoporous microspheres	744	50	552	[41]
ZnFe_2_O_4_ porous nanospheres	200	80	869	[45]
ZnMn_2_O_4_ microsheets	90	40	337	[49]
MgMn_2_O_4_ nanoparticles	50	100	484	[50]
Porous Zn_0.5_Mg_0.5_FeMnO_4_	150	80	300	This work

## Data Availability

Not applicable.

## Sample Availability

Samples of the Zn_0.5_Mg_0.5_FeMnO_4_ compound are available from the authors.
